# Effect of the Botanical Origin on Properties of RS3/4 Type Resistant Starch

**DOI:** 10.3390/polym11010081

**Published:** 2019-01-07

**Authors:** Tomasz Zięba, Małgorzata Kapelko-Żeberska, Artur Gryszkin, Aleksandra Wilczak, Bartosz Raszewski, Radosław Spychaj

**Affiliations:** 1 Department of Food Storage and Technology, Faculty of Food Science, Wroclaw University of Environmental and Life Sciences, Chełmońskiego 37, 51-630 Wrocław, Poland; tomasz.zieba@upwr.edu.pl (T.Z.); artur.gryszkin@upwr.edu.pl (A.G.); olawil@interia.pl (A.W.); bartosz.raszewski@upwr.edu.pl (B.R.); 2 Department of Fermentation and Cereals Technology, Faculty of Food Science, Wroclaw University of Environmental and Life Sciences, Chełmońskiego 37, 51-630 Wrocław, Poland; radoslaw.spychaj@upwr.edu.pl

**Keywords:** retrograded starch, biodiversity, acetylation, resistant starch

## Abstract

This study aimed to compare properties of retrograded starch acetates with an identical degree of substitution, but produced from raw materials of various botanical origin. Retrograded starch was produced from potato, wheat, corn, and tapioca starch, and afterwards acetylated with an acetic acid anhydride, adjusting reagent doses to achieve an identical degree of esterification of the modified preparation (2.1 g/100 g). Preparations of retrograded starch and acetylated retrograded starch differed significantly in their properties, which was due to the disparate botanical origin of starch. The highest susceptibility to acetylation was demonstrated for potato starch, and the lowest one for wheat starch. Acetylation of retrograded starch of various botanical origin increased its solubility in water, swelling power and viscosity of its pastes, as well as decreased its amylose content. Preparations of acetylated retrograded starches of disparate botanical origins may be deemed preparations of RS3/4 type resistant starch because they exhibit significant (23.5–34.0%) resistance to the activity of amylolytic enzymes.

## 1. Introduction

Intense investigations have been carried out within the last 30 years to identify production possibilities and properties of resistant starch, which is defined as products of its digestion not absorbed in the small intestine of a healthy man [1]. Resistant starch may be obtained by altering properties of the native starch via physical or chemical modification. One of the means to increase resistant starch content is the cold storage of products containing gelatinized starch. It leads to starch retrogradation, which is defined as the coupling of starch chains into ordered structures that are crystalline in character [2,3]. Properties of the resultant retrograded starch depend on, among other things, the origin and type of starch, conditions of the retrogradation process, and presence of other compounds [4,5,6,7,8]. Retrograded potato starch exhibits ca. 10% resistance to amylolysis [9]. The resistance to digestive enzymes may also be increased through chemical modifications of starch [3,10]. Acetylation of retrograded potato starch leads to its digestibility being reduced to ca. 60% [11], hence this starch may be deemed an RS3/4 type resistant starch (exhibiting properties of both RS3–retrograded starch and RS4–chemically-modified starch). Previous studies have focused on determining the properties of retrograded potato starch acetylated with various doses of acetic acid anhydride [9,12] and of acetylated retrograded potato starch crosslinked with various doses of adipic acid [13]. However, no research has been undertaken so far to compare properties of acetates of retrograded starch produced from starch of various origins.

This study aimed to compare properties of retrograded starch acetates with an identical degree of substitution, but produced from raw materials of various botanical origins.

## 2. Materials and Methods

### 2.1. Materials

Experimental materials included: Superior Standard potato starch manufactured by PEPEES Łomża S.A, wheat starch manufactured by Starch Production Plants of Grupa AWW Wawrzyniak (Kalisz, Poland), corn starch manufactured by Cargill Polska company (Wrocław, Poland), and tapioca starch produced in Bangkok, Thailand and distributed by EDMIR-POL company from Chorzów, Poland. Starch was acetylated with p.a. acetic acid anhydride purchased at POCH SA Gliwice company (Gliwice, Poland).

### 2.2. Preparation of Retrograded and Acetylated Starch

Native potato, wheat, corn, and tapioca starches were used to prepare starch pastes with the concentration of 10 g starch/100 g solution. Starch was kept in a water bath (Memmert, Germany) at a temperature of 94 °C for 6 h for complete gelatinization, then cooled and frozen at −20 °C for 72 h, and afterwards defrosted at 20 °C for 48 h. The produced starch having a spongy structure was disintegrated, rinsed with distilled water, dried in an air dryer (Memmert, Germany) at a temperature of 35 °C for 24 h, ground, and sieved through a screen with mesh size of 400 μm [9].

The manufactured preparations of retrograded starch were then subjected to the acetylation process according to the methodology provided in our previous work [14]. Doses of acetic acid anhydride used in the study were the nth-doses of acetic anhydride used in the acetylation process in the starch industry (13 cm^3^/100 g starch). The resultant preparations of acetylated retrograded starch were rinsed with distilled water, dried in an air dryer (Memmert, Germany) at a temperature of 30 °C for 24 h, ground, and sieved through a screen with a mesh size of 400 um.

### 2.3. Determination of the Content of Acetate Groups

The content of acetate groups was determined according to the methodology described by Wurzburg [15] and modified by Singh and Nath [16]. A suspension of 10 g of the preparation was prepared in 65 mL of water, then 0.1 M sodium chloride was instilled to the mixture to impart it with a light-pink color, which was maintained for one minute. Then, 25 cm^3^ of a 0.5 M NaOH solution were added to the mixture, which was shaken at a temperature of 25 °C for 35 min, and afterwards titrated with a 0.5 M solution of hydrochloric acid. The degree of esterification, expressed in grams of acid residues/100 g of the preparation, was computed from the following formula:

(1) $$A=\frac{\left(P_{0}-P_{w}\right)\cdot N_{k}0.043\cdot 100}{M}\;\;\left[g/100g\right]$$

where: *P*_0_—volume (mL) of titrated HCl solution used to titrate 25 mL of 0.5 mol/L NaOH, *P*_w_—volume (mL) of titrated HCl solution used to titrate the sample with acetylated starch, *N*_k_—acid titre, and *M*—content (g) of starch dry matter in the sample. 

### 2.4. Preparation of Retrograded and Acetylated Starch with an Identical Degree of Substitution

Based on the results achieved, the dependency between the degree of substitution and dose of acetic acid anhydride was determined for each type of starch. Afterwards, the analyzed starches were acetylated using doses of the reagent, ensuring the same degree of starch esterification in all modified preparations.

### 2.5. Determination of Amylose Content with Morrison’s Method

The content of amylose was determined with the iodometric method after dissolution of starch samples in a solution of dimethyl sulfoxide and urea. The absorbance was measured against a reference sample (not containing starch), 15 min after iodine solution was added, using a CECIL CE 2010 spectrophotometer (Cecil Instruments, London, UK) at a wavelength of 635 nm [17]. 

### 2.6. Determination of Swelling Power and Water Solubility at 80 °C

A water suspension of acetylated retrograded starch was prepared (which contained 1 g starch/100 g solution) and shaken at a temperature of 80 °C for 30 min. Afterwards, it was cooled to a temperature of 20 °C and centrifuged for 30 min in a Biofuge 28RS centrifuge (HeraeusSepatech, Hanau, Germany) at 22,500× *g*. Then, supernatant was collected and determined for dry matter content with the gravimetric method, whereas precipitate left in the centrifuge tubes was weighed [18]. 

### 2.7. Determination of Pasting Characteristics Using Brabender Viscograph

The pasting characteristics using the Brabender viscograph (Berlin, Germany) was conducted in a measuring vessel (type 700 cmg). A sample of starch preparation was transferred into a cylinder of the apparatus using 450 mL of distilled water, which resulted in a suspension containing 4 g starch per 100 g solution. The suspension was heated to 40 °C with a stirring rate of 75 rpm, and kept at this temperature for 10 min. Next, the contents of the cylinder were heated to 94 °C by 1.5 °C increments per min. The mixture was kept for 10 min at this temperature, then cooled at a rate of 1.5 °C per min to 30 °C, and kept at this temperature for another 10 min [19].

### 2.8. Determination of Resistance to the Activity of Amyloglucosidase

Suspensions were prepared that contained 0.36 g of starch per 100 g of the solution. They were heated to the boiling point at continuous mixing and, then, cooled to a temperature of 37 °C, at which hydrolysis with amyloglucosidase (Amigase by Genecor) was conducted. To the prepared suspension of a starch preparation, acetate buffer (pH = 4.5) was added at the ratio of 1:1. The flask was placed in a water bath with a shaker, at a temperature of 37 °C (Memmert, Schwabach, Germany), and 4 cm^3^ of an enzyme solution were added (at enzyme to buffer ratio of 1:4). The enzyme’s concentration was selected so as to enable complete saccharification of gelatinised native starch after 120 min of the process. Every hour, 1 cm^3^ of the hydrolysate was collected to centrifuge tubes that were centrifuged (MPW Instruments, Warsaw, Poland) with the speed of 1825× *g* for 5 min. Supernatant was collected from the centrifuged sample and mixed with a reagent from the Biosystem company (Glucose—containing glucose oxidase and peroxidase), and then incubated at 20 °C for 15 min. Afterwards, absorbance was measured using a CECIL CE 2010 colorimeter (Cecil Instruments) at the wavelength of λ = 500 nm. Measurements were conducted against a blank sample constituted by a reagent with an acetate buffer. The quantity of glucose was read out from the standard curve plotted as above using glucose (p.a.) solutions. The degree of saccharification was computed in respect of the theoretical quantity of glucose produced from the complete saccharification of a weighted portion of starch. The result of hydrolysis was final when three consecutive read outs of the absorbance did not differ between one another [9].

### 2.9. Statistical Analysis

To determine the effect of the botanical origin of starch on the values of the analyzed properties of the tested preparations, results achieved were subjected to a one-way analysis of variance at a significance level of *p* < 0.01. Based on statistical computations (from at least three parallel replications), values of the least significant differences (LSD) and standard deviation were calculated. Values of the least significant differences (LSD) were determined with a Duncan’s test at α = 0.05. Computations were performed using Statistica ver. 13.1 software (StatSoft, Inc., Tulsa, OK, USA, 2011). 

## 3. Results and Discussion

**Figure 1:**
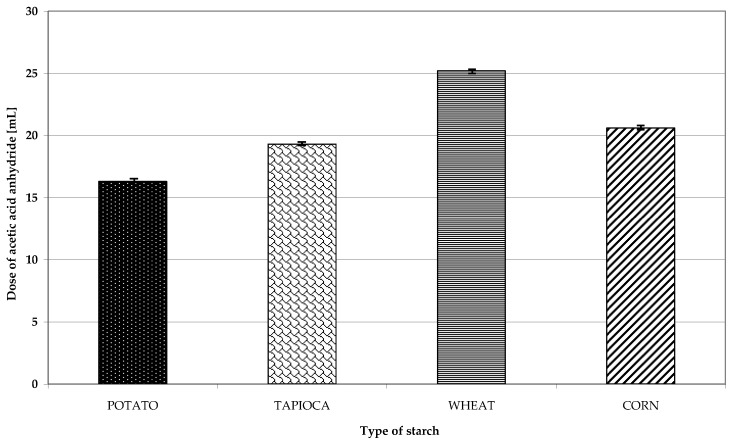
Dose of acetic acid anhydride needed to manufacture preparations of acetylated starch with an identical degree of substitution.

The outcome of starch acetylation is determined by many factors including, i.a., type and dose of reagent, temperature, medium pH, and reaction duration [20,21]. In the present study, retrograded starches of disparate botanical origin were acetylated to produce esters with an identical degree of substitution. It turned out that the resultant preparations exhibited various susceptibility to acetylation. To achieve a degree of substitution at 2.1%, the lowest dose of acetic acid anhydride (16.3 mL) was used during acetylation of retrograded potato starch. More reagent was needed in the case of retrograded tapioca, maize, and wheat starches—i.e., 18%, 26%, and 55%, respectively (Figure 1). Different susceptibility to acetylation exhibited by starches from various botanical sources has been reported by many authors. Mbougueng et al. [22] acetylated potato and tapioca starches, whereas Singh et al. [23] acetylated corn and potato starches. In both cases, it was the potato starch that turned out to be more susceptible to esterification with acetic acid anhydride. Different observations were made by Gunaratne and Corke [24], as in their study, the most susceptible to acetylation was wheat starch followed by potato and corn starches. The most frequently mentioned factors claimed to affect native starch susceptibility to acetylation include: Size and internal structure of starch granules, in particular the arrangement of crystalline and amorphous structures. Worthy of notice is that we acetylated retrograded starch whose susceptibility to acetylation depends not only on starch origin, but also on the concentration of the paste the retrograded starch is made of [9].

**Figure 2:**
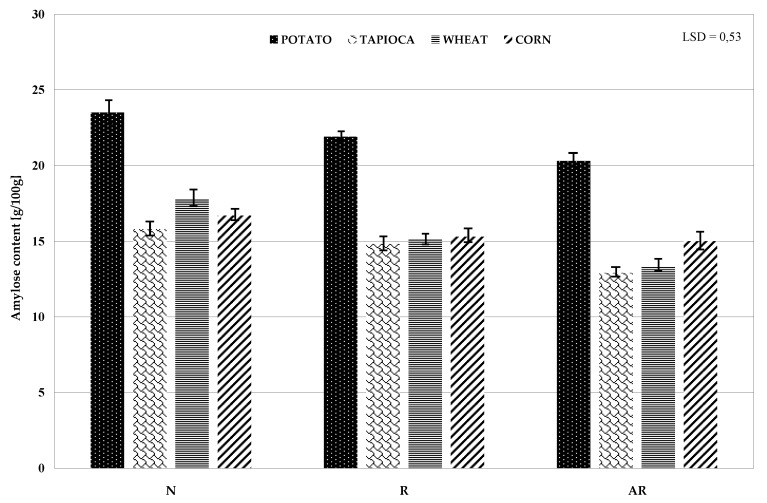
Contents of amylose in native (N), retrograded ®, and acetylated retrograded (AR) starch of various botanical origins.

The key factor which differentiates properties of starch is its botanical origin [25]. Amylose content of starch is also affected by multiple factors, like the starch fraction size [26,27] or method and conditions of starch material cultivation [28,29], and in the case of retrograded starch, also the concentration of frozen paste [9,14]. In the present study, native potato starch contained much more amylose compared to the other analyzed starches, and retrogradation decreased amylose content in all manufactured preparations (Figure 2). Most likely, retrogradation of amylose (folding into double helices) occurred during starch paste freezing only when amylose chains were in direct contact with each other. In our study, we froze 10% paste, which, after cooling, formed a rigid gel. Single simple chains of amylose entrapped in branched amylopectin might not be retrograded, and after defrosting, were removed with water as a soluble fraction. In accordance with literature data, chemical modifications of starch may affect its amylose content. Some authors have reported acetylated starch to contain more amylose than the native starch [22,30,31], while others have suggested just the opposite [19,32,33]. In turn, Akintayo et al. [34] and Huang et al. [35] showed no significant differences in contents of amylose and amylopectin between the analyzed starches. In our study, acetylation decreased amylose content in all tested preparations. 

**Figure 3:**
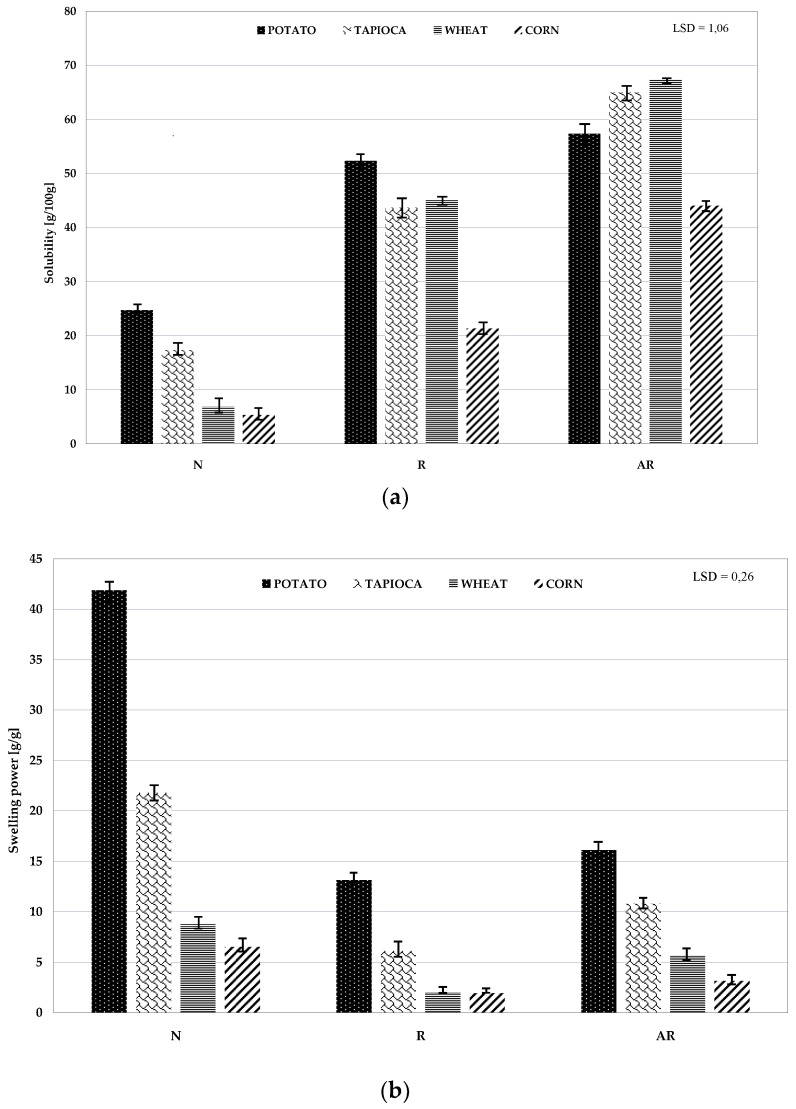
Solubility (**a**) and swelling power (**b**) of native (N), retrograded ®, and acetylated retrograded (AR) starch of various botanical origin.

Native starches of different botanical origins exhibit various solubility in water and swelling power. The highest swelling power has been reported for potato starch, whereas the swelling power of cereal starches is lower due to their different crystalline structure and the complexation of the part of their amylose chains with lipids’ substances, which reduces their water absorption capability [36]. Figure 3 depicts solubility in water and the swelling power of native, retrograded, and acetylated retrograded starches of various botanical origins. The retrograded starch produced by freezing and defrosting of starch pastes exhibited a lower swelling power and a higher solubility in water compared to the native starch. Presumably, ice crystal formed during freezing causes partial damage to the structure of amylose and amylopectin chains. Many authors have previously demonstrated changes in the values of solubility in water and swelling power during the manufacture of different types of modified starch preparations [37,38,39,40]. Acetylation of retrograded starch caused a significant increase in both the swelling power and solubility of the analyzed preparations. Native starch acetates with a low degree of substitution are more soluble in water and have a higher swelling power compared to the native starches [22,35].

**Figure 4:**
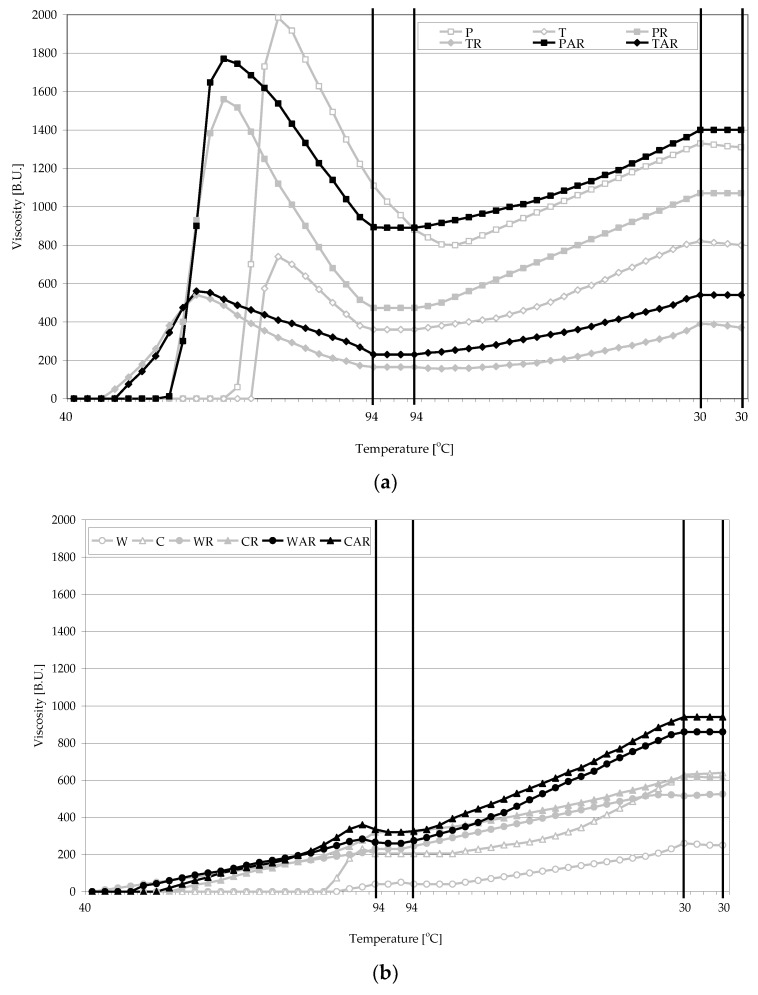
Brabender pasting characteristics of native (N), retrograded ®, and acetylated retrograded (AR) starch from: (**a**)—potato and tapioca, (**b**)—wheat and corn.

Figure 4 presents Brabander pasting characteristics determined for starches of various botanical origins as well as for retrograded and acetylated retrograded preparations made of them. Pastes made of native starches from different botanical sources are characterized by diversified viscosity, which has been extensively documented in the literature [41,42,43]. In our study, the highest paste viscosity (maximal and determined at a temperature of 30 °C) was determined for potato starch (i.e., 1985 and 1330 B.U., respectively), followed by tapioca starch (740 and 820 B.U.), corn starch (230 and 635 B.U.), and wheat starch (40 and 255 B.U.). Pastes produced from retrograded potato and tapioca starches exhibited lower viscosity (1540, 1040 and 540, 390 B.U., respectively), those made of corn starch exhibited similar viscosity (230, 620 B.U.), whereas those produced from wheat starch exhibited higher viscosity (230, 520 B.U.). Gryszkin et al. studied the effect of freezing heated starch suspensions of various botanical origins and demonstrated a positive correlation between the size of the formed structures of retrograded starch and the viscosity of the starch pastes. In the case of retrograded potato starch, the viscosity of the formed pastes decreases, whereas in the case of wheat starch, it increases significantly compared to the pastes produced from native starch [44,45,46]. In all samples, acetylation of retrograded starches increased the paste viscosity (to 1740, 1400 B.U. in the case of potato starch, to 230, 520 B.U. in the case of tapioca starch, to 360, 940 B.U. in the case of corn starch, and to 285, 860 B.U. in the case of wheat starch) compared to the pastes made of retrograded starches. Acetylation of starch may either increase or decrease paste viscosity as affected by many factors, just to mention the method and extent of acetylation or the type of raw material [21].

**Figure 5:**
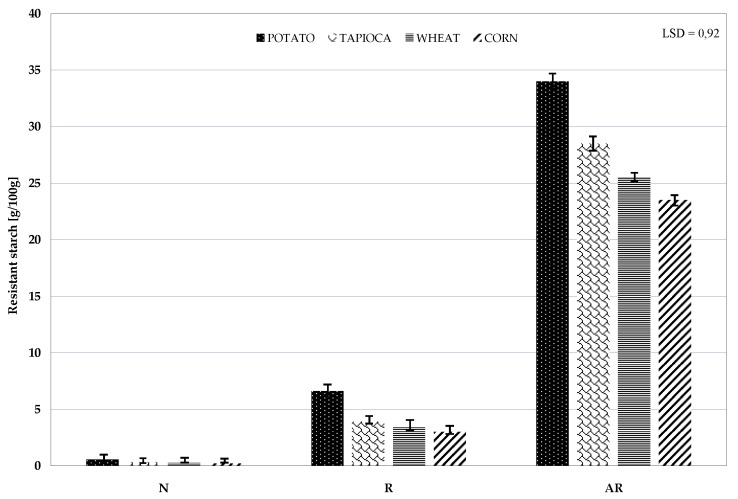
Content of resistant starch in native (N), retrograded ®, and acetylated retrograded (AR) starch of various botanical origins.

Disparity in the structure resulting from the botanical origin and common modifications of starch, which alter the spatial structure of the starch chain, cause its partial resistance to the activity of digestive enzymes. Garcia-Alonso et al. investigated the effect of the botanical origin and of the method of the pastes’ production from wheat, corn, rice, and potato starches on the content of their resistant starch fraction [47]. They demonstrated that both the type of starch and the process of retrogradation contributed to a change in starch susceptibility to the activity of digestive enzymes. Differences in the susceptibility of starch preparations from various sources to these enzymes have also been confirmed by other authors [48,49]. Figure 5 presents the results of determinations of the susceptibility of the manufactured preparations to amylolysis. The pasted native starch was almost completely saccharified by amyloglucosidase. Retrogradation increased preparations’ resistance to amylolysis, which ranged from 3.0% to 6.6% depending on the starch type. In turn, preparations of acetylated retrograded starches exhibited high resistance (23.5–34.0%), which is, presumably, due to the coupling of the resistance imparted by retrogradation and then by acetylation, which was shown in our previous research [9,14]. The differences in the resistance of acetylated retrograded starch of various botanical origins were most likely due to disparities in the structure of individual starches (e.g., length of non-branched chains in amylopectin or presence of amylose-lipid complexes in cereal starch). These structural differences could affect not only the aforementioned susceptibility of starch to acetylation, but also the site of acetyl group substitution. In our earlier work, we have demonstrated that ester substitution at the 2nd and 3rd atom of carbon had a greater impact on the starch resistance increase than the substitution at the 6th atom of carbon of anhydroglucose [19].

## 4. Conclusions

The botanical disparity of native starch determined differences in properties of the manufactured preparations of retrograded starch and acetylated retrograded starch with an identical degree of substitution. Among the starches subjected to one-stage modification, the greatest susceptibility to acetylation, the highest content of amylose, the highest swelling power, solubility in water, viscosity of formed pastes, and resistance to amylolysis was demonstrated for retrograded potato starch, whereas the lowest values of the above parameters were shown for wheat starch. Acetylation of retrograded starches of various botanical origins enhanced all their studied properties except for the amylose content, which decreased irrespective of the raw material origin. Preparations of acetylated retrograded starches of various botanical origins may be deemed preparations of RS3/4 type resistant starch because they exhibit significant (23.5–34.0%) resistance to the activity of amylolytic enzymes.
